# Inter-Individual Diversity Scaling Analysis of the Human Virome With Classic Diversity-Area Relationship (DAR) Modeling

**DOI:** 10.3389/fgene.2021.627128

**Published:** 2021-04-20

**Authors:** Wanmeng Xiao, Zhanshan (Sam) Ma

**Affiliations:** ^1^Computational Biology and Medical Ecology Laboratory, State Key Laboratory of Genetic Resources and Evolution, Kunming Institute of Zoology, Chinese Academy of Sciences, Kunming, China; ^2^Kunming College of Life Sciences, University of Chinese Academy of Sciences, Kunming, China; ^3^Center for Excellence in Animal Evolution and Genetics, Chinese Academy of Sciences, Kunming, China

**Keywords:** human virome ecology, diversity scaling, diversity-area relationship, potential diversity, maximal accrual diversity

## Abstract

The human virome is a critical component of the human microbiome, and it is believed to hold the richest diversity within human microbiomes. Yet, the inter-individual scaling (changes) of the human virome has not been formally investigated to the best of our knowledge. Here we fill the gap by applying diversity-area relationship (DAR) modeling (a recent extension to the classic species-area law in biodiversity and biogeography research) for analyzing four large datasets of the human virome with three DAR profiles: DAR scaling (*z*)—measuring the inter-individual heterogeneity in virome diversity, *MAD* (maximal accrual diversity: *D*_*max*_) and *LGD* ratio (ratio of local diversity to global diversity)—measuring the percentage of individual to population level diversity. Our analyses suggest: (*i*) The diversity scaling parameter (*z*) is rather resilient against the diseases as indicated by the lack of significant differences between the healthy and diseased treatments. (*ii*) The potential maximal accrual diversity (*D*_*max*_) is less resilient and may vary between the healthy and diseased groups or between different body sites. (*iii*) The *LGD* ratio of bacterial communities is much smaller than for viral communities, and relates to the comparatively greater heterogeneity between local vs. global diversity levels found for bacterial-biomes.

## Introduction

Viruses spread over almost every ecosystem and are believed to be the most abundant biological entity on the earth (Breitbart and Rohwer, 2005; Edwards and Rohwer, 2005; Cobiaìn Güemes et al., 2016; Berliner et al., 2018). Viruses also parasitize in different parts of human body, including blood, cerebrospinal fluid, nasal cavity, oral cavity, skin, vagina, lungs and gastrointestinal (GI) tract (Nakamura et al., 2009; Ly et al., 2014; Wylie et al., 2014; Hannigan et al., 2015; Santiago-Rodriguez et al., 2015; Columpsi et al., 2016; Abbas et al., 2017; Thannesberger et al., 2017; Pannaraj et al., 2018; Ghose et al., 2019). The human virome includes endogenous retroviruses, eukaryotic viruses that infect human cells, bacteriophages that infect bacteria, and viruses that infect archaea (Zhao et al., 2017; Santiago-Rodriguez and Hollister, 2019). In addition to the well-known human health effects of the human immunodeficiency virus (HIV), Ebola virus, and influenza virus, changes in the composition and diversity of virome have been found in many diseases, such as cystic fibrosis, periodontal disease, urinary tract infections, and inflammatory bowel disease (Willner et al., 2009; Ly et al., 2014; Norman et al., 2015; Santiago-Rodriguez et al., 2015; Carding et al., 2017). Bacteriophages can also affect human health by influencing the composition of the bacterial communities in the body (Barr et al., 2013; Carding et al., 2017; Galtier et al., 2017; Hannigan et al., 2018). In addition, phages were briefly used to treat bacterial infections in humans prior to the inventions of antibiotics; today phage therapy is still confined to laboratory and animal models (Debarbieux et al., 2013). In summary, the influences of viruses on human health are not limited to the their pathogenicity. There are complex interactions and even co-evolution between viruses and hosts (Parker, 2016).

But why have the studies of human virome, a vital part of human microbiomes, been behind those of bacteria and fungi? Different from prokaryotes and eukaryotes, the virome does not encode commonly conservative genes, and is highly diversified in genes (Carding et al., 2017). However, with the development of high-throughput sequencing technology, metagenomic sequencing and the application of many effective bioinformatics approaches, human virome studies have stepped into an era of rapid development (Reyes et al., 2012; Garmaeva et al., 2019). The Global Virome Project (GVP) launched recently (Carroll et al., 2018) is aimed to identify and characterize most of the currently unknown viruses in major wildlife populations, including rodents, non-human primates and bats. A number of studies have shown that human gut virome is stable in individuals over a certain period of time, but shows high heterogeneity among individuals (Minot et al., 2011, 2013; Carding et al., 2017; Clooney et al., 2019; Shkoporov et al., 2019).

Here we apply the diversity-area relationship (DAR) model, an extension of the classical species-area relationship (SAR) model, to measure the diversity changes (scaling) of human viral communities from multiple perspectives (Watson, 1835; Arrhenius, 1921; Preston, 1960, 1962; Ma, 2018, 2019). Specifically, four diversity-scaling profiles were used to characterize the spatial (inter-individual) heterogeneity, community similarity, potential diversity, and the ratio of local diversity to global accrual diversity of human virome in health and disease.

## Materials and Methods

### Datasets Description

We collected four human virome datasets from NCBI (Table 1). Data-1 consists of 147 bronchoalveolar lavage samples taken from lung donors and recipients in the United States, and 34 blood serum samples taken by lung transplant recipients. The bronchoalveolar lavage samples contain 80 PGD (Primary Graft Dysfunction) samples and 67 control samples, the blood serum samples contain 18 PGD samples and 16 control samples. The feces sample of Data-2 were from healthy Amerindian children in four different places in Venezuela, including 20 samples from Urban A, 10 samples from Village B, 16 samples from Village C, and 15 samples from Village D. Data-3 consisted of 10 IBD patients and five healthy subjects from Canada, including six CD (Crohn’s disease) samples, four UC (ulcerative colitis) samples, and five Control samples. Data-4 is a collection of stool samples collected from the Hadza Hunter-gatherers of Tanzania and covering five sub-seasons, including 30 matched viral and bacterial sample.

**TABLE 1:** A brief description on the virome datasets reanalyzed in this study*.

**Dataset**	**BioProject**	**SRA**	**Sample size**		**Material**	**Strategy**	**References**
			**Original data**	**VirusSeeker**			
Data-1	PRJNA390659	SRP109620	35	34	Blood serum	WGA	Abbas et al., 2017 *Am J Transplant*
			160	147	Bronchoalveolar lavage	WGA/Other	
Data-2	PRJNA418044	SRP124915	80	61	Feces	WGS	Siqueira et al., 2018 *Nat Commun*
Data-3	PRJNA421331	SRP126261	16	15	Colon	RNA-Seq	Wang et al., 2015 *Inflamm Bowel Disease*
Data-4	PRJNA392180	SRP110665	40	30	Stool	WGS	Smits et al., 2017 *Science*
	PRJNA392012	ERP109605	1,305	30		16S rRNA	

*The first three datasets are virome only and the fourth dataset includes both virome and bacterial samples.

### Bioinformatics Pipeline for Bacterial Sequences

All raw sequences were processed by QIIME2 v2018-06 (Bolyen et al., 2018) pipeline to get the OTU (operational taxonomic unit) tables. Sequences were denoised by the DADA2 plugin and taxonomic classification was performed using the Greengenes database and QIIME feature-classifier classify-sklearn plugin.

### Bioinformatics Pipeline for Viral Sequences

Analysis of all viral sequences was based on the VirusSeeker-Virome pipeline (Zhao et al., 2017), included the following 10 steps: (1) Extract the SRA data using the fastq-dump tool of the NCBI SRA Toolkit v2.9.2^1^; (2) Stitch read1 and read2 together using fastq-join in the ea-utils package^2^ (Aronesty, 2011); (3) Sequences quality control using PRINSEQ v0.20.4 (Schmieder and Edwards, 2011); (4) Run seqtk to convert fastq format to fasta format^3^; (5) For further sequences quality control, run Tantan v13 (Frith, 2011) and RepeatMasker v2.1^4^ (Smit et al., 2013–2015) to screen interspersed repeats and low complexity DNA sequences; (6) Remove human sequences by aligning sequences to reference genome (GRCh37/hg19) using BWA-MEM v0.7.11 (Li and Durbin, 2010; *L* = 100,100; *k* = 15) and MegaBLAST (ncbi-blast v2.6.0+, *E*-value = 1e^–9^); (7) Input remaining reads to blastn (ncbi-blast v2.6.0+, *E*-value = 1e^–2^) alignment against the virus-only nucleotide database and blastx (ncbi-blast v2.6.0+, *E*-value = 1e^–2^) against the virus-only protein database; (8) The leftover candidate eukaryotic viral sequences were sequentially searched and mapped against the NCBI Bacteria reference genomes using BWA-MEM (-*k* = 15), NT database using MegaBLAST (*E*-value = 1e^–8^), blastn (*E*-value = 1e^–8^), and NCBI NR database using blastx (*E*-value = 1e^–8^); (9) Got a summary including “phage,” “ambiguous,” “unassigned,” and “assignment”; (10) Generated viral OTU tables based on assignment report.

### DAR (Diversity-Area Relationship) Analysis

DAR analysis is implemented through the DAR model, involving the PL (power law) model and the PLEC (power law with exponential cutoff) model (Ma, 2018, 2019). The DAR model extended the classical ecological power law of species-area relationship (SAR), by replacing the species richness (number of species or OTUs) in the classic law with more general community diversity metrics measured in Hill Numbers (Chao et al., 2012, 2014a,b).

The relationship between diversity and area conform to the power law function:

(1) Dq=c⁢Az

where *^*q*^D* represents the diversity of measured by Hill numbers when order is *q*, *A* is area, and *c* and *z* are parameters of the power law scale model. In addition, Ma extended the general power law to an exponential cutoff power law scale model (Ma, 2018, 2019), and the PLEC model was initially applied to SAR modeling (Plotkin et al., 2000; Ulrich and Buszko, 2003; Tjørve, 2009):

(2) Dq=c⁢Az⁢exp⁢(d⁢A)

where *d* is the third parameter of the power law equation, and exp(*dA*) is the exponential decay term.

After log-linear transformation of the above power law equation, which can be used to estimate DAR model parameters:

(3) ln⁢(D)=ln⁢(c)+z⁢ln⁢(A)

(4) ln⁢(D)=ln⁢(c)+z⁢ln⁢(A)+d⁢A

**FIGURE 1:**
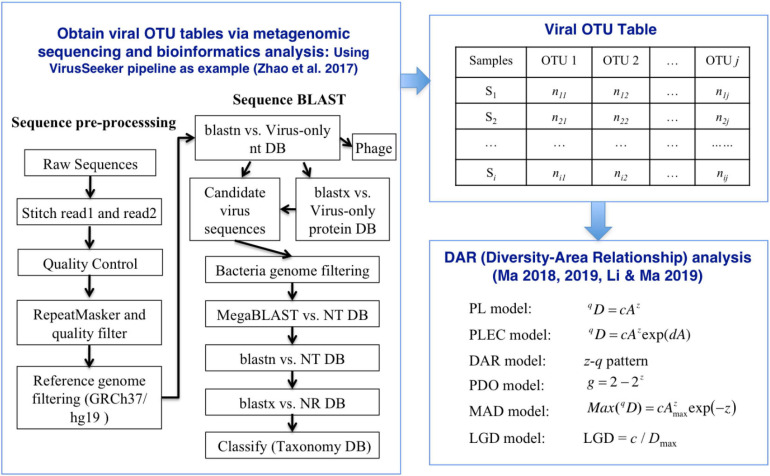
The process of inter-individual diversity scaling analysis of human virome with diversity-area relationship (DAR) modeling.

When no natural order exists among samples, or the order (permutation) information is not available, the choice of an arbitrary permutation may be problematic. To avoid this potential bias, we re-sampled all the virome and bacterial datasets, that is, we randomly selected 100 permutations after permutation of all the samples in each dataset to fit the DAR model, and finally used the average parameters of the 100 models as the final DAR model. Meanwhile, the goodness-of-fitting can be judged based on the linear correlation coefficient *R* and *p*-value. Ma (2018) also defined four diversity-scaling profiles based on the DAR model:

(*i*) *DAR* profile: The relationship between diversity scaling parameter (*z*) and diversity order (*q*) of DAR-PL model was defined as *DAR* profile.

(*ii*) *PDO* (pair-wise diversity overlap) profile: The relationship between parameter g and diversity order (*q*) of DAR-PL model was defined as *PDO* profile. Parameter *g* measures the diversity overlap of two areas,

(5) $$g=2-2^{z}$$

where *z* is the parameters in equation (1).

(*iii*) *MAD* (maximum accrual diversity) profile: The *MAD* can be derived from the following equation based on the parameter of DAR-PLEC model:

(6) M⁢a⁢x⁢(Dq)=Dmaxq=c⁢(-zd)z⁢exp⁢(-z)=c⁢Amaxz⁢exp⁢(-z)

where _*A_max =–z/d*_, which represents the area when the diversity is maximized. The relationship between *MAD* and the diversity order (*q*) was defined as *MAD* Profile:

(*iv*) *LGD* (the ratio of local diversity to global accrual diversity) profile: Li and Ma (2019) defined the ratio of sample diversity to global diversity as *LGD*:

(7) $$\text{LGD}=c/D_{\text{max}}$$

where *c* is the parameter of Equations (1) at *q*-th diversity order, and *D*_*max*_ corresponding to the diversity order can be obtained by equation (6). The relationship between *LGD* and the diversity order was defined as *LGD* profile. To test whether there are significant differences in DAR parameters between different subsets in the four virus datasets, we performed a permutation (randomization) test (Ma, 2018; Li and Ma, 2019; Ma and Li, 2019). Figure 1 displayed the workflow of the VirusSeeker pipeline and subsequent steps for DAR analysis using the viral OTU table.

## Results

### Fitting DAR Models

After an initial bioinformatics analysis of four datasets, we fitted the DAR-PL and DAR-PLEC models to the three datasets of virome datasets and one bacteria-virome dataset. Table 2 shows the changes in the number of viral sequences during data processing, and Table 3 lists all parameters related to model fitting. Table 4 gives the results of the permutation test for three virome datasets. Although the four datasets cover different disease types, sampling sites, and microbial types, the results show that all datasets confirmed to the DAR-PL and DAR-PLEC models, with *p-*value < 0.05. After 100 re-sampling for the four datasets, data-1, data-2, data-4 and the CD group of data-3 fully followed the DAR-PL pattern at the species richness level (*q* = 0), but some groups were not fitted successfully in the higher diversity order. At higher diversity orders, some model fittings failures occurred.

**TABLE 2:** Summary of changes in the number of viral sequences*.

**Dataset**	**Material**	**Total Num. of sequences**	**QC**	**RP**	**Ref Filtered**
Data-1	Blood serum	17,455,532	731,028	446,528	204,093
	Bronchoalveolar lavage	129,172,831	49,717,378	31,838,935	28,841,071
Data-2	Feces	23,982,017	16,249,374	14,950,131	13,279,027
Data-3	Colon	16,805,869	16,805,869	7,833,742	15,773,053
Data-4	Stool	165,351,779	130,551,328	128,817,549	128,283,393

*QC, number of sequence remaining after quality control; RP, number of sequence remaining after RepeatMasker; RefFiltered, number of sequence remaining after filtering the human reference genome.

**TABLE 3:** Fitting the DAR (diversity-area relationship) models (with 100 times of random permutations of samples) for the four virome datasets.

**Dataset**	**Group**	**Diversity order**	**Power law (PL)**						**PL with exponential cutoff (PLEC)**								
			***z***	**ln (*c***)	***R***	***p*-value**	***g***	***N***	***z***	***d***	**ln(*c*)**	***R***	***p*-value**	***N***	***A*_*max*_**	***D*_*max*_**	***LGD***
Data-1	Blood-Control-LTR	*q* = 0	0.949	2.589	0.926	0.000	0.019	100	1.467	–0.089	2.392	0.953	0.000	71	51.766	304.1	0.044
		*q* = 1	–0.009	1.145	0.756	0.007	0.975	62	0.294	–0.053	1.081	0.803	0.007	63	12.852	3.8	0.818
		*q* = 2	–0.028	0.744	0.748	0.007	0.998	67	-0.028	–0.002	0.785	0.821	0.005	65	10.036	2.1	1.002
		*q* = 3	–0.031	0.641	0.767	0.005	1.002	62	-0.057	0.004	0.664	0.823	0.006	68	15.983	1.8	1.027
	Blood-PGD-LTR	*q* = 0	0.815	2.999	0.939	0.000	0.208	100	1.086	–0.039	2.852	0.964	0.000	60	29.266	236.1	0.085
		*q* = 1	–0.171	1.250	0.738	0.005	1.081	49	-0.413	0.045	1.330	0.788	0.006	71	12.880	2.4	1.464
		*q* = 2	–0.149	0.803	0.718	0.004	1.090	66	-0.301	0.026	0.863	0.807	0.004	76	38.758	1.6	1.401
		*q* = 3	–0.128	0.658	0.723	0.004	1.079	71	-0.251	0.020	0.713	0.812	0.004	76	19.150	1.4	1.337
	Lung-Control-LTR	*q* = 0	0.687	3.918	0.867	0.000	0.347	100	1.423	–0.058	3.010	0.915	0.000	81	46.017	512.1	0.098
		*q* = 1	0.295	2.742	0.788	0.000	0.763	100	0.682	–0.033	2.315	0.885	0.000	90	25.547	41.4	0.375
		*q* = 2	0.143	2.111	0.666	0.003	0.892	91	0.413	–0.024	1.840	0.797	0.001	87	20.862	13.8	0.600
		*q* = 3	0.108	1.887	0.653	0.004	0.920	81	0.346	–0.021	1.662	0.779	0.001	86	25.281	9.9	0.666
	Lung-Control-OD	*q* = 0	0.653	4.144	0.884	0.000	0.378	100	1.366	–0.066	3.398	0.932	0.000	83	73.294	513.4	0.123
		*q* = 1	0.329	2.655	0.816	0.000	0.730	100	0.638	–0.029	2.333	0.884	0.000	85	30.034	41.5	0.343
		*q* = 2	0.225	1.991	0.762	0.001	0.826	96	0.411	–0.018	1.818	0.842	0.001	82	38.755	14.8	0.496
		*q* = 3	0.192	1.773	0.718	0.003	0.854	92	0.361	–0.017	1.629	0.806	0.002	81	63.514	10.8	0.546
	Lung-PGD-LTR	*q* = 0	0.814	3.544	0.866	0.000	0.189	100	1.414	–0.051	2.922	0.915	0.000	84	57.413	611.5	0.057
		*q* = 1	0.341	2.541	0.808	0.000	0.724	100	0.669	–0.027	2.183	0.884	0.000	90	53.661	40.5	0.313
		*q* = 2	0.174	1.978	0.689	0.003	0.869	91	0.406	–0.021	1.752	0.813	0.001	89	793.901	13.3	0.542
		*q* = 3	0.154	1.739	0.676	0.003	0.886	81	0.356	–0.019	1.578	0.791	0.002	88	21.213	9.8	0.582
	Lung-PGD-OD	*q* = 0	0.669	4.026	0.881	0.000	0.375	100	1.252	–0.042	3.282	0.933	0.000	86	40.907	599.5	0.094
		*q* = 1	0.280	2.691	0.810	0.000	0.776	100	0.595	–0.022	2.268	0.870	0.000	83	34.851	39.3	0.376
		*q* = 2	0.131	2.026	0.617	0.003	0.901	84	0.325	–0.015	1.809	0.730	0.001	79	35.447	12.4	0.614
		*q* = 3	0.104	1.808	0.603	0.004	0.922	77	0.282	–0.014	1.621	0.709	0.001	76	38.125	9.2	0.666
Data-2	Urban A	*q* = 0	0.463	5.150	0.954	0.000	0.614	100	0.781	–0.042	4.912	0.975	0.000	83	53.386	713.8	0.242
		*q* = 1	0.388	2.021	0.814	0.002	0.681	88	0.397	–0.006	2.098	0.870	0.001	64	21.716	21.4	0.353
		*q* = 2	0.355	1.333	0.798	0.003	0.713	89	0.298	0.005	1.434	0.850	0.002	57	18.375	9.1	0.415
		*q* = 3	0.345	1.130	0.788	0.003	0.723	88	0.251	0.011	1.233	0.840	0.003	54	16.284	7.0	0.443
	Village B	*q* = 0	0.524	5.212	0.951	0.000	0.537	100	1.120	–0.134	5.028	0.979	0.000	74	14.836	579.1	0.317
		*q* = 1	0.509	2.382	0.872	0.006	0.564	79	0.804	–0.081	2.407	0.929	0.005	67	16.783	37.6	0.288
		*q* = 2	0.583	1.303	0.899	0.004	0.490	62	0.713	–0.053	1.468	0.918	0.008	58	176.180	32.2	0.114
		*q* = 3	0.550	1.082	0.887	0.006	0.524	64	0.661	–0.040	1.193	0.926	0.006	50	13.203	10.2	0.289
	Village C	*q* = 0	0.368	5.591	0.956	0.000	0.702	100	0.665	–0.048	5.427	0.980	0.000	85	28.545	716.4	0.374
		*q* = 1	0.285	2.218	0.754	0.007	0.750	50	0.085	0.016	2.535	0.790	0.008	72	20.631	22.1	0.416
		*q* = 2	0.345	1.253	0.771	0.006	0.707	38	-0.155	0.050	1.913	0.758	0.011	61	7.020	7.6	0.459
		*q* = 3	0.347	0.977	0.754	0.010	0.712	37	-0.190	0.060	1.611	0.776	0.008	54	6.771	5.6	0.472
	Village D	*q* = 0	0.380	5.597	0.960	0.000	0.683	100	0.700	–0.055	5.439	0.982	0.000	86	25.876	726.1	0.371
		*q* = 1	0.342	2.876	0.884	0.001	0.725	99	0.437	–0.016	2.820	0.925	0.001	70	35.503	41.6	0.427
		*q* = 2	0.367	2.105	0.872	0.001	0.704	98	0.416	–0.010	2.097	0.913	0.001	72	23.848	20.1	0.408
		*q* = 3	0.370	1.847	0.859	0.001	0.701	98	0.436	–0.014	1.843	0.906	0.001	65	274.037	18.0	0.353
Data-3	Control	*q* = 0	0.817	3.872	0.951	0.015	-0.002	59	2.293	–0.638	4.607	0.984	0.032	40	3.895	190.7	0.252
		*q* = 1	0.963	1.162	0.913	0.030	0.000	12	3.353	–0.997	2.089	0.979	0.042	9	3.333	16.5	0.194
		*q* = 2	0.248	1.468	0.929	0.024	0.775	31	1.231	–0.349	1.568	0.983	0.033	31	3.592	6.7	0.652
		*q* = 3	0.329	1.127	0.916	0.030	0.726	31	1.362	–0.376	1.253	0.985	0.030	21	3.815	5.2	0.591
	CD	*q* = 0	0.540	5.177	0.962	0.003	0.453	100	1.136	–0.204	5.242	0.988	0.004	75	16.212	454.7	0.390
		*q* = 1	0.361	3.275	0.912	0.015	0.694	51	1.006	–0.215	3.318	0.976	0.012	47	5.197	48.4	0.546
		*q* = 2	0.520	1.847	0.955	0.004	0.565	26	0.989	–0.214	2.202	0.977	0.012	28	5.176	16.1	0.393
		*q* = 3	0.496	1.500	0.951	0.004	0.589	26	0.902	–0.184	1.811	0.979	0.011	26	4.940	10.7	0.418
	UC	*q* = 0	0.490	5.393	0.975	0.025	0.576	87	0.795	–0.079	5.279	0.999	0.037	17	32.210	1,227.9	0.179
		*q* = 1	0.249	3.398	0.988	0.012	0.812	35	0.249	–0.069	3.673	1.000	0.021	12	2.546	42.0	0.712
		*q* = 2	0.183	2.359	0.989	0.011	0.865	27	0.840	–0.343	2.821	1.000	0.012	6	3.054	15.8	0.670
		*q* = 3	0.133	2.067	0.986	0.014	0.903	27	0.719	–0.309	2.493	1.000	0.015	6	2.934	11.0	0.715
Data-4	Bacteria	*q* = 0	0.704	4.203	0.969	0.000	0.360	100	1.017	–0.030	3.883	0.980	0.000	78	126.016	861.2	0.078
		*q* = 1	0.448	3.274	0.890	0.000	0.625	100	0.736	–0.029	3.023	0.937	0.000	91	52.102	120.9	0.219
		*q* = 2	0.310	2.387	0.755	0.001	0.753	84	0.540	–0.029	2.319	0.823	0.001	82	20.767	29.9	0.364
		*q* = 3	0.241	2.081	0.693	0.005	0.811	83	0.469	–0.028	1.979	0.813	0.001	80	49.833	18.3	0.438
	Virome	*q* = 0	0.081	6.373	0.972	0.000	0.942	100	0.117	–0.004	6.340	0.988	0.000	95	68.970	767.4	0.763
		*q* = 1	0.007	3.394	0.742	0.003	0.995	70	0.012	–0.001	3.394	0.786	0.002	79	30.873	30.5	0.978
		*q* = 2	0.000	2.229	0.681	0.004	1.000	74	0.002	0.000	2.229	0.778	0.001	74	22.955	9.3	0.998
		*q* = 3	0.001	1.962	0.692	0.003	1.000	81	0.003	0.000	1.961	0.794	0.002	77	22.476	7.1	0.997

**TABLE 4:** The *p*-values of the permutation tests for the differences in the parameters of the DAR models between each pair-wise comparison of three virome datasets.

**Diversity order**	**Dataset**	**Comparison of groups**	**PL**		**PLEC**					
			***z***	**ln (*c*)**	***z***	***d***	**ln (*c*)**	***A*_*max*_**	***D*_*max*_**	***LGD***
*q* = 0	Data-1	Blood-Control-LTR vs. Blood-PGD-LTR	0.774	0.775	0.716	0.715	0.744	0.526	0.567	0.732
		Blood-Control-LTR vs. Lung-Control-LTR	0.692	0.491	0.965	0.835	0.709	0.919	0.383	0.570
		Blood-PGD-LTR vs. Lung-PGD-LTR	0.999	0.741	0.780	0.927	0.968	0.563	0.341	0.685
		Lung-Control-LTR vs. Lung-PGD-LTR	0.812	0.820	0.985	0.891	0.968	0.608	0.476	0.676
		Lung-Control-OD vs. Lung-PGD-OD	0.978	0.940	0.905	0.704	0.954	0.379	0.606	0.773
		Lung-Control-LTR vs. Lung-Control-OD	0.951	0.897	0.950	0.912	0.833	0.384	0.995	0.829
		Lung-PGD-LTR vs. Lung-PGD-OD	0.763	0.780	0.860	0.856	0.865	0.578	0.934	0.675
	Data-2	Urban A vs. Village B	0.821	0.926	0.578	0.324	0.867	0.316	0.413	0.716
		Urban A vs. Village C	0.688	0.466	0.825	0.906	0.480	0.450	0.975	0.449
		Urban A vs. Village D	0.725	0.438	0.865	0.802	0.450	0.426	0.915	0.477
		Village B vs. Village C	0.544	0.512	0.481	0.394	0.529	0.550	0.224	0.814
		Village B vs. Village D	0.550	0.485	0.487	0.399	0.497	0.602	0.203	0.803
		Village C vs. Village D	0.939	0.987	0.930	0.878	0.982	0.841	0.883	0.989
	Data-3	Control vs. CD	0.690	0.523	0.601	0.534	0.648	0.236	0.275	0.716
		Control vs. UC	0.764	0.512	0.593	0.638	0.645	0.049	0.032	0.791
		CD vs. UC	0.873	0.680	0.732	0.729	0.948	0.235	0.036	0.619
	Percentage (%) with significant differences		0	0	0	0	0	6.3% (1/16)	12.5% (2/16)	0
*q* = 1	Data-1	Blood-Control-LTR vs. Blood-PGD-LTR	0.660	0.898	0.429	0.413	0.765	0.998	0.357	0.395
		Blood-Control-LTR vs. Lung-Control-LTR	0.633	0.322	0.734	0.907	0.455	0.614	0.134	0.449
		Blood-PGD-LTR vs. Lung-PGD-LTR	0.417	0.444	0.419	0.681	0.603	0.095	0.044	0.250
		Lung-Control-LTR vs. Lung-PGD-LTR	0.870	0.818	0.986	0.872	0.895	0.326	0.888	0.823
		Lung-Control-OD vs. Lung-PGD-OD	0.875	0.969	0.937	0.855	0.954	0.859	0.706	0.918
		Lung-Control-LTR vs. Lung-Control-OD	0.915	0.926	0.953	0.919	0.986	0.753	0.979	0.919
		Lung-PGD-LTR vs. Lung-PGD-OD	0.810	0.858	0.878	0.835	0.929	0.507	0.781	0.864
	Data-2	Urban A vs. Village B	0.759	0.707	0.669	0.633	0.770	0.791	0.257	0.907
		Urban A vs. Village C	0.796	0.855	0.709	0.852	0.693	0.937	0.973	0.913
		Urban A vs. Village D	0.879	0.326	0.960	0.914	0.423	0.563	0.287	0.868
		Village B vs. Village C	0.631	0.876	0.514	0.690	0.887	0.593	0.429	0.820
		Village B vs. Village D	0.580	0.435	0.574	0.548	0.559	0.353	0.839	0.685
		Village C vs. Village D	0.871	0.474	0.696	0.821	0.755	0.321	0.338	0.979
	Data-3	Control vs. CD	0.554	0.163	0.352	0.353	0.425	0.587	0.196	0.601
		Control vs. UC	0.552	0.210	0.371	0.484	0.369	0.758	0.284	0.510
		CD vs. UC	0.690	0.827	0.362	0.591	0.432	0.471	0.536	0.647
	Percentage (%) with significant differences		0	0	0	0	0	0	6.3% (1/16)	0
*q* = 2	Data-1	Blood-Control-LTR vs. Blood-PGD-LTR	0.638	0.931	0.644	0.736	0.894	0.124	0.507	0.443
		Blood-Control-LTR vs. Lung-Control-LTR	0.721	0.275	0.643	0.879	0.411	0.613	0.135	0.487
		Blood-PGD-LTR vs. Lung-PGD-LTR	0.547	0.370	0.507	0.737	0.466	0.005	0.078	0.418
		Lung-Control-LTR vs. Lung-PGD-LTR	0.871	0.788	0.988	0.887	0.899	0.009	0.885	0.854
		Lung-Control-OD vs. Lung-PGD-OD	0.628	0.950	0.831	0.903	0.993	0.877	0.400	0.757
		Lung-Control-LTR vs. Lung-Control-OD	0.714	0.851	0.997	0.857	0.980	0.404	0.702	0.793
		Lung-PGD-LTR vs. Lung-PGD-OD	0.792	0.924	0.830	0.765	0.928	0.008	0.706	0.853
	Data-2	Urban A vs. Village B	0.523	0.971	0.661	0.747	0.971	0.036	0.015	0.572
		Urban A vs. Village C	0.982	0.927	0.574	0.718	0.581	0.434	0.834	0.924
		Urban A vs. Village D	0.979	0.341	0.856	0.894	0.406	0.794	0.268	0.983
		Village B vs. Village C	0.604	0.957	0.467	0.676	0.630	0.015	0.006	0.576
		Village B vs. Village D	0.484	0.209	0.689	0.727	0.356	0.054	0.188	0.476
		Village C vs. Village D	0.956	0.340	0.557	0.720	0.813	0.246	0.222	0.928
	Data-3	Control vs. CD	0.753	0.741	0.905	0.839	0.612	0.664	0.251	0.683
		Control vs. UC	0.947	0.549	0.862	0.997	0.400	0.843	0.222	0.975
		CD vs. UC	0.386	0.391	0.873	0.696	0.262	0.451	0.943	0.475
	Percentage (%) with significant difference		0	0	0	0	0	31.3% (5/16)	6.3% (1/16)	0
*q* = 3	Data-1	Blood-Control-LTR vs. Blood-PGD-LTR	0.657	0.983	0.692	0.827	0.931	0.702	0.509	0.476
		Blood-Control-LTR vs. Lung-Control-LTR	0.756	0.273	0.641	0.847	0.381	0.641	0.131	0.525
		Blood-PGD-LTR vs. Lung-PGD-LTR	0.565	0.371	0.530	0.758	0.427	0.871	0.084	0.440
		Lung-Control-LTR vs. Lung-PGD-LTR	0.762	0.721	0.977	0.927	0.879	0.772	0.964	0.795
		Lung-Control-OD vs. Lung-PGD-OD	0.631	0.944	0.832	0.873	0.991	0.345	0.434	0.757
		Lung-Control-LTR vs. Lung-Control-OD	0.663	0.834	0.957	0.888	0.963	0.179	0.673	0.760
		Lung-PGD-LTR vs. Lung-PGD-OD	0.739	0.877	0.825	0.761	0.942	0.422	0.741	0.803
	Data-2	Urban A vs. Village B	0.549	0.951	0.653	0.773	0.957	0.837	0.524	0.742
		Urban A vs. Village C	0.994	0.852	0.598	0.708	0.637	0.470	0.809	0.931
		Urban A vs. Village D	0.935	0.364	0.789	0.836	0.424	0.032	0.155	0.863
		Village B vs. Village C	0.652	0.906	0.478	0.685	0.634	0.388	0.516	0.763
		Village B vs. Village D	0.559	0.226	0.758	0.835	0.344	0.036	0.317	0.853
		Village C vs. Village D	0.946	0.307	0.530	0.663	0.761	0.021	0.116	0.836
	Data-3	Control vs. CD	0.833	0.731	0.794	0.747	0.630	0.739	0.379	0.761
		Control vs. UC	0.815	0.443	0.757	0.930	0.363	0.737	0.272	0.815
		CD vs. UC	0.304	0.306	0.848	0.701	0.216	0.460	0.924	0.430
	Percentage (%) with significant differences		0	0	0	0	0	18.8% (3/16)	0	0

### DAR Profiles

**FIGURE 2:**
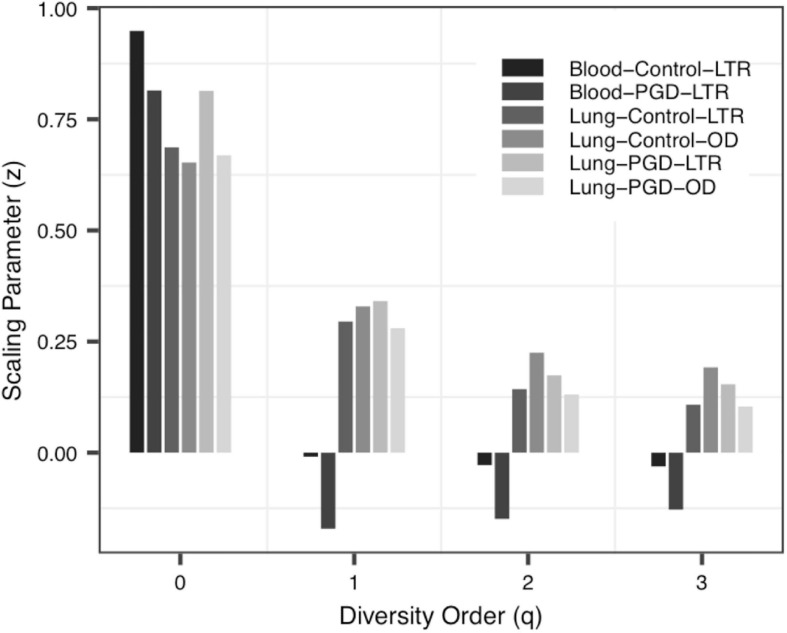
The DAR (diversity-area relationship) profiles of Data-1.

**FIGURE 3:**
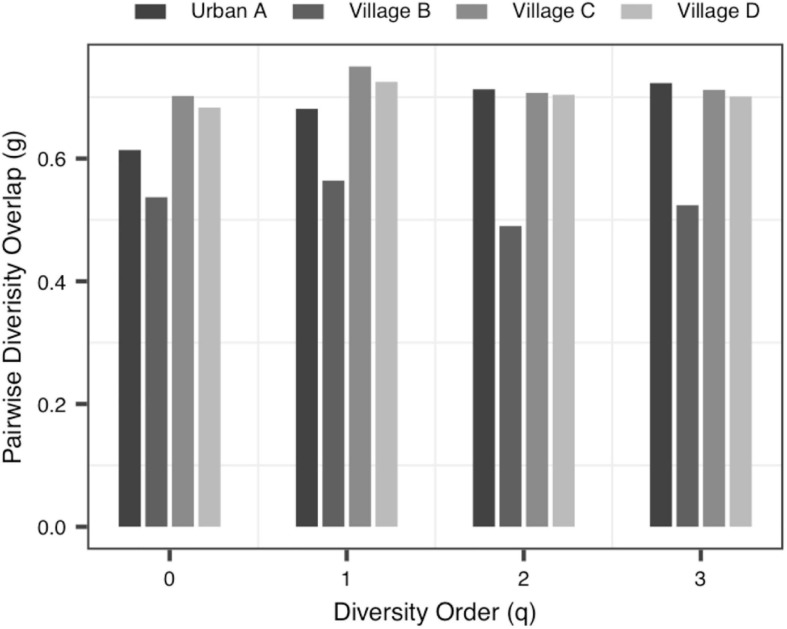
The PDO (pair-wise diversity overlap) profiles of Data-2.

**FIGURE 4:**
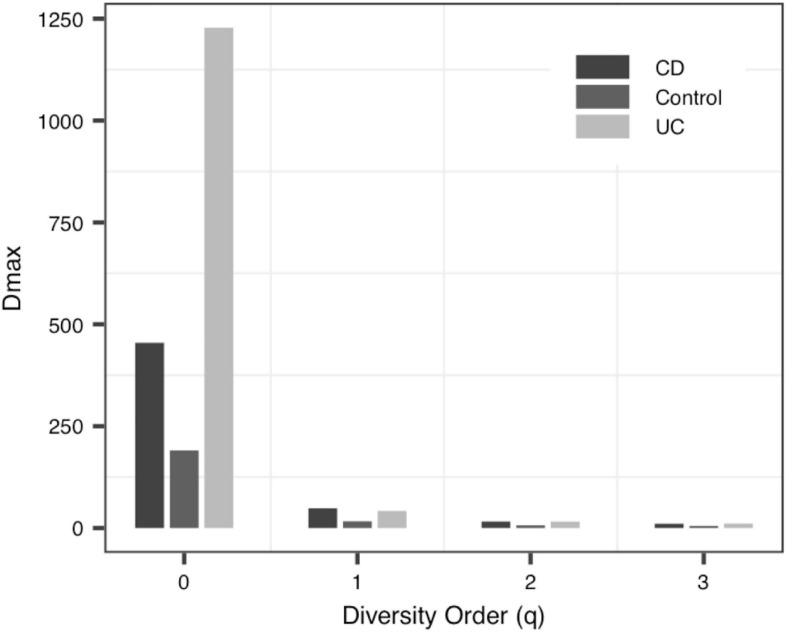
The MAD (maximal accrual diversity) profiles of Data-3.

**FIGURE 5:**
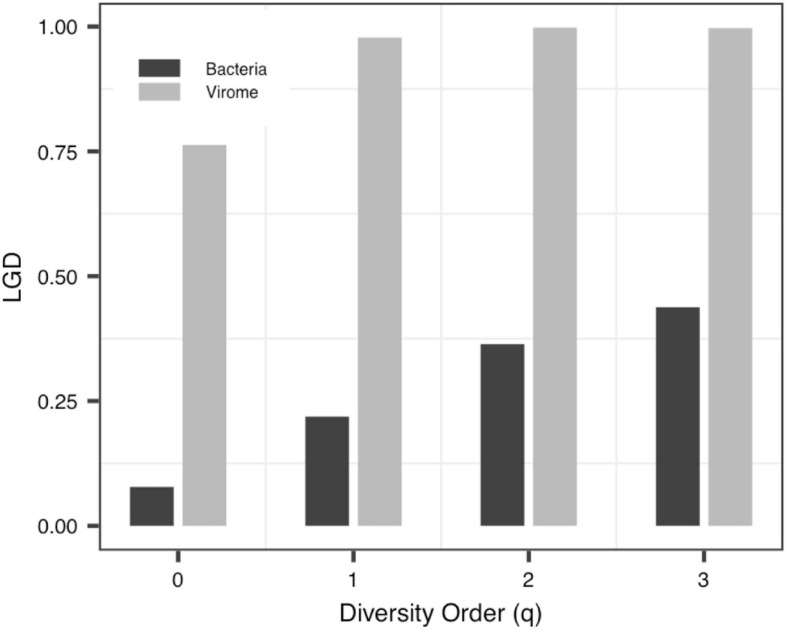
The LGD (ratio of local to global diversity) profiles of Data-4.

With the exception of a few groups, the patterns of the four *DAR* profiles across the four datasets are consistent in general (Table 3). Data-1 and Data-3 were the comparison between the healthy group and the disease group. Although the disease types were different, the variation patterns of the DAR parameters are similar in general. The results in Table 4 show that there was no significant difference in the diversity scaling parameter (*z*) and the pair-wise diversity overlap (*g*) among three virome datasets. However, the estimators of the maximal accrual diversity (*D*_*max*_) between the Control group and the UC group and between the CD group and the UC group presented significant difference when *q* = 0. In addition, the results of Data-4 show that bacterial community and virome community had different *LGD* ratios. And based on Table 3, we also have the following findings:

(i) The *DAR* profile: The parameter of diversity scaling (*z*) of Data-1 (the Blood-Control-LTR group: *z* = 0.949, −0.009, −0.028, −0.031; the Lung-Control-LTR group: *z* = 0.687, 0.295, 0.143, 0.108; the Lung-Control-OD group: *z* = 0.653, 0.329, 0.225, 0.192; the Lung-PGD-LTR group: *z* = 0.814, 0.341, 0.174, 0.154; the Lung-PGD-OD group: *z* = 0.669, 0.280, 0.131, 0.104), the Urban A group (*z* = 0.463, 0.388, 0.355, 0.345) of Data-2, the UC group (*z* = 0.490, 0.249, 0.183, 0.133) of Data-3 and the bacteria group (*z* = 0.704, 0.448, 0.310, 0.241) of Data-4 all decreased with the rise of the diversity order, Figure 2 shows the downward trend of parameter *z* in Data-1. But the parameter *z* of the Blood-Control-LTR group in Data-1, the Village C and Village D group in Data-2 were the largest when *q* = 0, and had the minimum value when *q* = 1, and then enlarge with the increase of diversity order (*q* > 0). When the diversity order *q* = 0, the difference in species number among individuals in each group was measured; When *q* > 0, the *DAR* profile measured the difference in the diversity of dominant species and the number of each species among individuals in each group. With the increase of the diversity order, the proportion of dominant species in the calculation process increased. These suggested that at the lower level of diversity, the higher heterogeneity within the group, indicating the greater difference between individuals within the group. It also should be noted that there was no significant difference in the parameter *z* among all groups, even between the healthy and diseased groups.

(ii) The *PDO* profile: In all datasets, the pair-wise diversity overlap (*g*) of more than half of the groups grew with the increase of the order of diversity. Although the *g* varies among individuals with different tissue types, different health conditions and different living environments, there is no significant difference. And the larger the *g* was, the higher the degree of overlap was. Thus, the difference between the individuals within the group at the low diversity order was greater than that at the high diversity order (*q* = 0 − 1), Figure 3 shows the variation of *g* in Data-2.

(iii) The *MAD* Profile: The estimators of the *MAD* of four datasets reduced with the increase of the diversity order. When *q* = 0, that is, at the species richness level, *D*_*max*_ represents the estimated maximum number of species, and the *A*_*max*_ represents the number of individuals required to reach the maximum number of species. In Data-1, the *MAD*s in the alveolar samples of donors and recipients are very similar, but with some differences between the control group and the PGD group at the species richness level. Simultaneously, there was no significant difference in *MAD* between the bacteria group and the virome group in Data-4. As for Data-3, the *MAD* of the control group and the CD group were significantly smaller than the UC group at *q* = 0 (Figure 4).

(iv) The *LGD* profile: In addition to the Blood-PGD-LTR group in Data-1, the Village B and the Village D group in Data-2, Data-3 and the virome group in Data-4, the *LGD* ratios in other groups increased with the rise of the diversity order. In the first three datasets, the *LGD* ratios of the same sample type are relatively close, such as Village C vs. Village D (0.374 vs. 0.371, *q* = 0); Lung-control-LTR vs. Lung-control-OD (0.098 vs. 0.123, *q* = 0). According to Data-4, we can see that bacteria and virome have completely different *LGD* ratios (0.078 vs. 0.763, *q* = 0). Figure 5 displays the growth of *LGD* in Data-4 and the differences between the bacteria group and the virome group in *LGD*.

## Conclusions and Discussion

To the best of our knowledge, this should be the first application of DAR analysis to the human virome, which offers a powerful tool to investigate the spatial (inter-individual) variability (heterogeneity) of virome diversity, and potential virome diversity globally (in a whole population). With the *MAD* Profile, *D*_*max*_ estimates the maximum number of viral species in the community when diversity order *q* = 0. It can be seen that, in Data-1, which includes blood samples and alveolar samples of the donors and the lung transplant recipients, the maximum number of species in blood serum samples was lower than that in bronchoalveolar lavage samples, and this may indicate that the species diversities of different tissues is diverse (Ghose et al., 2019). At the same time, there was no statistically significant difference between the four profiles in the bronchoalveolar lavage samples of the donors and the recipients, perhaps because the virome of donor was transferred to the recipient along with the organ transplant (Mitchell and Glanville, 2018; Abbas et al., 2019). Data-2 compared the virome of one urban and three villages children. And we did not observe significant differences in the scaling parameter *z* and the pair-wise diversity overlap *g* among four locations, especially Village C and Village D. Thus, differences in living environment and diet do not seem to have a significant impact on the spatial heterogeneity of individual gut virome. For data-3, we can clearly see that when *q* = 0, the scaling parameter *z* of the control group is greater than that of the two disease groups CD and UC, and the pair-wise diversity overlap *g* of the control group is much smaller than that of the CD group and UC group. The changes of these two profiles suggest that there was a large difference among individuals in the Control group, while the CD and UC groups show a smaller heterogeneity among individuals. In addition, the *D*_*max*_ of the UC group was significantly greater than that of the Control and CD group, and the virome diversity of the CD and UC group was significantly increased. These findings are also consistent with existing research showing increased virome diversity in IBD patients compared to healthy individuals (Lepage et al., 2008; Norman et al., 2015; Wang et al., 2015). In IBD patients, the diversity of viruses increased while the diversity of bacteria decreased (Norman et al., 2015; Clooney et al., 2019; Fernandes et al., 2019; Zuo et al., 2019). We also compared bacteria and virome in stool samples from hunter-gatherer Hadza people living in Tanzania. Based on the *MAD* profiles, it was revealed that the virome community and the bacteria community have similar maximum number of species. However, according to the *LGD* profiles, the ratios of local diversity to global diversity are quite different between virome and bacterial community. The differences may be due to limitations in virome analysis methods (including biochemical and bioinformatics methods), as well as to the limited virome library available (Aggarwala et al., 2017; Santiago-Rodriguez and Hollister, 2019).

Although this study is the first to compare the diversity scaling parameters between the healthy and diseased treatments, due to the very limited number of datasets, we are refrained from inferring general conclusions about possible effects of diseases on the inter-individual diversity scaling. We hope that future studies will allow us to draw more general conclusions on this important topic. As a very preliminary step, our analysis seems to suggest that the disease effects on the inter-individual diversity scaling (or the inter-individual heterogeneity of diversity) may be insignificant. Nevertheless, our study demonstrates an important quantitative tool (i.e., DAR) for analyzing the inter-individual heterogeneity of virome diversity, which is of obvious significance for investigating the ecology of human virome.

## Data Availability Statement

The original contributions presented in the study are included in the article/supplementary material, further inquiries can be directed to the corresponding author/s.

## Ethics Statement

Ethical review and approval was not required for the study on human participants in accordance with the local legislation and institutional requirements. Written informed consent for participation was not provided by the participants’ legal guardians/next of kin because the datasets we used are published data.

## Author Contributions

ZM designed the study and revised the manuscript. WX performed the data analysis and wrote the draft. Both authors approved the submission.

## Conflict of Interest

The authors declare that the research was conducted in the absence of any commercial or financial relationships that could be construed as a potential conflict of interest.

## Footnotes

1 http://www.ncbi.nlm.nih.gov/Traces/sra/

2 https://github.com/ExpressionAnalysis/ea-utils

3 https://github.com/lh3/seqtk.git

4 http://www.repeatmasker.org
